# Supplementary material for: Predicting and forecasting the impact of local outbreaks of COVID-19: use of SEIR-D quantitative epidemiological modelling for healthcare demand and capacity
Source: Int J Epidemiol. 2021 Jul 9;50(4):1103–13. doi: 10.1093/ije/dyab106 (PMC8407866; doi:10.1093/ije/dyab106)
Supplement: dyab106_Supplementary_Data [file dyab106_supplementary_data.pdf]

Supplementary Material

Predicting and forecasting the impact of local outbreaks of COVID-19: Use of SEIR-D quantitative epidemiological modelling for healthcare demand and capacity

Eduard Campillo-Funollet*[1], James Van Yperen[2], Phil Allman[3], Michael Bell[4], Warren Beresford[5], Jacqueline Clay[6], Matthew Dorey[6], Graham Evans[7], Kate Gilchrist[4], Anjum Memon[8], Gurprit Pannu[9], Ryan Walkley[6], Mark Watson[10], and Anotida Madzvamuse[2]

[1]School of Life Sciences, Centre for Genome Damage and Stability, University of Sussex, Brighton, BN1 9QH, UK
[2]School of Mathematical and Physical Sciences, Department of Mathematics, University of Sussex, Brighton, BN1 9QH, UK
[3]NHS Sussex Commissioners, Wicker House, Worthing, BN11 1DJ, UK
[4]Public Health Intelligence and Adult Social Care, Brighton and Hove City Council, 2nd Floor, Hove Town Hall, Norton Road, Hove, BN3 3BQ, UK
[5]Planning and Intelligence, Brighton and Hove, Sussex Commissioners, East Sussex, UK
[6]Public Health and Social Research Unit, West Sussex County Council, 1st Floor, The Grange, Tower Street, Chichester, West Sussex, PO19 1RG, UK
[7]Public Health Intelligence, East Sussex County Council, St Annes Crescent, Lewes, BN7 1UE, UK
[8]Department of Primary Care and Public Health, Brighton and Sussex Medical School, Brighton, BN1 9QH, UK
[9]Sussex Health and Care Partnership, First Floor, Millview Hospital, Nevill Avenue, Hove, East Sussex, BN3 7HY, UK
[10]Sussex Health and Care Partnership, 36-38 Friars Walk, Lewes, BN7 2PB, UK

1 Description of Mathematical Model

We propose a novel model which breaks down the typical infectious compartment of a Susceptible-Exposed-Infectious-Recovered-Dead (SEIR-D) model into two compartments, one strand to model individuals who become infected with COVID-19 and will be going to hospital, and the other strand to model individuals who won't need to go to hospital and thus remain undetected by healthcare requirements. The objective of separating the strands like this is so we can fit the model to available data presented to us by the Sussex Clinal Commissioning Group (CCG) and local County Council authorities (Brighton & Hove, East and West Sussex) using our novel inference algorithm detailed in Sections 2.1 and 2.2.

The mathematical model takes the form of a temporal epidemiological dynamic system of ordinary differential equations governed by the interactions depicted in Figure 1 supported by non-negative

*Corresponding author: E.Campillo-Funollet@sussex.ac.uk

initial conditions:

$$\begin{aligned}
\dot{S} &= -\beta\frac{U+I}{N}S, && t\in(0,T], && S(0)=S_0, && (1)\\
\dot{E} &= \beta\frac{U+I}{N}S-\gamma_E E, && t\in(0,T], && E(0)=E_0, && (2)\\
\dot{U} &= p\,\gamma_E E-\gamma_U U, && t\in(0,T], && U(0)=U_0, && (3)\\
\dot{I} &= (1-p)\gamma_E E-\gamma_I I, && t\in(0,T], && I(0)=I_0, && (4)\\
\dot{H} &= \gamma_I I-(\gamma_H+\mu_H)H, && t\in(0,T], && H(0)=H_0 && (5)\\
\dot{R}_U &= (1-m_U)\gamma_U U, && t\in(0,T], && R_U(0)=R_{U,0}, && (6)\\
\dot{R}_H &= \gamma_H H, && t\in(0,T], && R_H(0)=R_{H,0}, && (7)\\
\dot{D}_U &= m_U\gamma_U U, && t\in(0,T], && D_U(0)=D_{U,0}, && (8)\\
\dot{D}_H &= \mu_H H, && t\in(0,T], && D_H(0)=D_{H,0}. && (9)
\end{aligned}$$

In this setting, let N denotes the total regional population in the Sussex regions of the UK (with N approximately 1.7 million). Then, $S(t)$ denotes the proportion of the total population N who are susceptible to the disease, COVID-19. These become exposed to the disease, i.e. they are carrying the disease but are not currently infectious, to form the $E(t)$ subpopulation at rate $\lambda(t)$ which represents the current infectivity. The rate $\lambda(t)$ is the product between β, the average transmission rate, and the probability that a contact to an individual leads to an infection $(U(t)+I(t))N^{-1}$. The $E(t)$ subpopulation is in an incubation compartment and individuals become infectious at an average rate γ_E. A proportion of $E(t)$ becomes infectious but, in spirit of the healthcare demand, remain undetected, denoted $U(t)$, with probability p. The other proportion of $E(t)$ becomes infectious and will require hospitalisation in the future, denoted $I(t)$, with probability $1-p$. The subpopulation that does not require hospitalisation can either progress to recover, at an average rate γ_U with probability $1-m_U$, to form the recovered population, denoted by $R_U(t)$, or die, at an average rate γ_U with probability m_U, to form the dead population, denoted by $D_U(t)$. Considering the infectious population that will be going to hospital, these individuals will become hospitalised, denoted by $H(t)$, and thus be in hospital care at an average rate γ_I. Once in hospital, patients can evolve in two separate pathways, a proportion of the hospitalised population can fully recover at an average rate γ_H to form the subpopulation denoted by $R_H(t)$. Alternatively, if they can not recover, then they die whilst in hospital at an average rate μ_H, to form the dead population denoted by $D_H(t)$.

In the spirit of epidemiological models of this nature, β denotes the average transmission rate, γ_E^{-1} denotes the average incubation time, p denotes the average proportion of infectious individuals who will not require hospital treatment, γ_U^{-1} denotes the average infectious period, m_U denotes the average infected fatality ratio for undetected cases, γ_I^{-1} denotes the average infectious period from becoming infectious to being admitted to hospital, γ_H^{-1} denotes the average hospitalisation period for those who recover and μ_H^{-1} represents the average hospitalisation period for those who die.

2 Methodology for parameter inference

Now that we have formulated the full model system to be studied, we next present the methodology for parameter inference given reliable datasets as mentioned in the previous section. Indeed, we were presented with hospital data, made up of daily hospital admissions (the red dashed arrow between $I(t)$ and $H(t)$ in Figure 1), daily hospital discharges (the red dashed arrow between $H(t)$ and $R_H(t)$ in Figure 1) and daily hospital bed occupancy (the red dashed $H(t)$ compartment in Figure 1), as well as weekly death data, which was split into weekly hospital deaths (the blue double dashed arrow between $H(t)$ and $D_H(t)$ in Figure 1) and weekly outside of hospital deaths (the red dashed arrow between $U(t)$ and $D_U(t)$ in Figure 1). We note here that the weekly death data outside of hospital is dominated by deaths in care homes.

In this section we will go into the mathematical details of the inference algorithm described in the **Inferring model parameters given hospital datasets** section of the manuscript. The algorithm

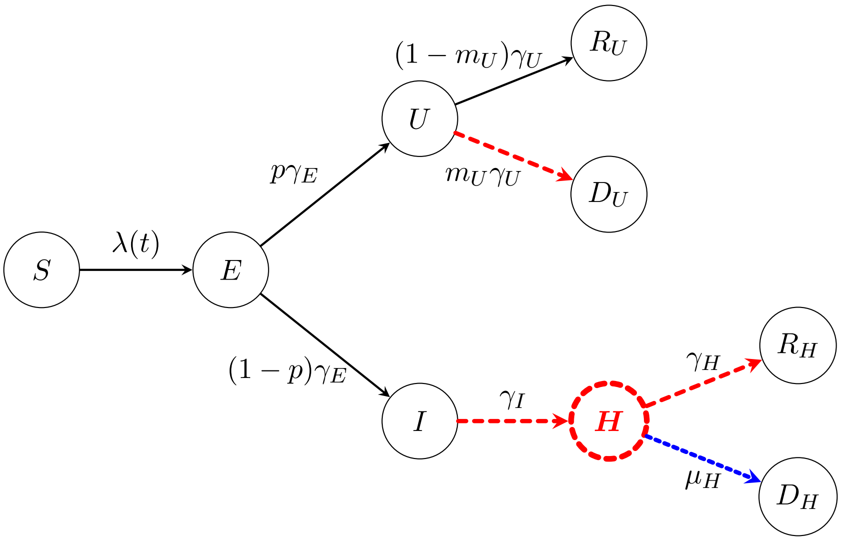

Figure 1: Schematic representation of the compartment model.

is two fold. First we exploit the linear relationships between mortality in hospitals and discharges from hospitals. Second we derive the observational model, a representation of equations (1)–(5) described only in terms of the model parameters and compartments that are captured by the mathematical interpretation of the data, and use an inverse approach to fit that to data using the maximum likelihood estimate. These two methods combined give us values for all the model parameters and initial conditions.

2.1 Regression analysis to infer hospitalisation discharge and death rates

2.1.1 Mathematical notation

Given that we have hospital discharge data and those that die in hospital, we start by considering (5), where we describe the number of individuals moving from the hospital compartment $H(t)$ to the hospital recovered $R_H(t)$ and then we describe the number of individuals moving from the hospital compartment $H(t)$ to the death in hospital compartment $D_H(t)$. We then seek to find a linear relationship between the two descriptions and use linear regression analysis to estimate the relationship between them.

We begin by considering the hospital discharged data which is available for 84 consecutive days starting March 24th, 2020. We denote the set of data points by C_H, with each data point denoted as $C_{H,i}$, for $i = 1, \dots, 84$. By considering (5) and (7), the rate of individuals being discharged in a given day from hospital is given by $\gamma_H H$. This allows us to compute the number of discharges per day in the following way

$$c_H(t) := \gamma_H \int_{t-1}^{t} H(s)\,\mathrm{d}s, \quad t \geq 1, \tag{10}$$

where t is a given day. We assume that the data is perturbed with Gaussian noise of unknown mean, denoted $\bar{x}_H$, and unknown standard deviation, denoted σ_H, which leads to the following relationship

$$C_{H,i} = c_H(i) + \bar{x}_H + \xi_{H,i}, \tag{11}$$

where i denotes a time point of the data and $\xi_{H,i} \sim \mathcal{N}(0, \sigma_H^2)$.

We now consider the data for those who die in hospitals. The data for deaths in hospitals is available on a weekly basis, starting on week 14 (week ending April 3rd, 2020) up to week 24 (week ending June 12th, 2020). We denote the set of data points by $C^w_{D_H}$, with each data point denoted as $C^w_{D_H,j}$, for $j = 14, \dots, 24$. By considering (5) and (9), the rate of individuals dying in hospital in a given day is given by $\mu_H H$. In a similar fashion, we can calculate the number of deaths per day in

the following manner

$$c_{D_H}(t) := \mu_H \int_{t-1}^{t} H(s)\,\mathrm{d}s, \quad t \geq 1. \tag{12}$$

Since the data is weekly rather than daily, the number of deaths per week is calculated by

$$c^w_{D_H}(\tau) := \mu_H \int_{\tau-7}^{\tau} H(s)\,\mathrm{d}s, \quad \tau \geq 7\,(\text{weekly}). \tag{13}$$

We again assume that the data is perturbed with Gaussian noise of unknown mean, denoted by $\bar{x}_D$, and unknown standard deviation, denoted by σ_D, which leads to the following relationship

$$C^w_{D_H,j} = c^w_{D_H}(t_j) + \bar{x}_D + \xi_{D,j}, \tag{14}$$

where t_j is the last day of week j and $\xi_{D,j} \sim \mathcal{N}(0, \sigma_D^2)$.

2.1.2 Regression analysis

From (10) and (12) we find

$$c_H(t) = \frac{\gamma_H}{\mu_H} c_{D_H}(t), \tag{15}$$

which is a consequence of equations (5), (7) and (9), and gives light to one of the parameters, $\gamma_H \mu_H^{-1}$, to be estimated. We are going to treat the daily deaths as unknowns in our model, by solving the least squares problem defined by (11) and (14), together with

$$c^w_{D_H}(\tau) = \sum_{k=0}^{6} c_{D_H}(\tau - k).$$

For simplicity, we assume that the covariance for the weekly mortality data is seven times larger than the covariance for the daily discharged data, i.e. $\sigma_D^2 = 7\sigma_H^2$. The rationale of this assumption is that the relationship assumes that on the same time scale, mortality and discharged data would have the same noise levels, and that mortality data is composed of independent daily measurements. We note that this is a conservative assumption, since data collection methods are diverse.

Let $\eta = \gamma_H \mu_H^{-1}$. The corresponding negative log-likelihood for the model given by (11) and (14) is

$$F(\eta, \bar{x}_H, \bar{x}_D, c_{D_H}; C_H, C^w_{D_H}) := \frac{1}{|\mathcal{I}|} \sum_{i=1}^{84} \left(\eta\, c_{D_H}(i) + \bar{x}_H - C_{H,i}\right)^2 + \frac{1}{7|\mathcal{J}|} \sum_{j=14}^{23} \left(\sum_{k=0}^{6} c_{D_H}(t_j - k) + \bar{x}_D - C^w_{D_H,j} \right)^2, \tag{16}$$

where $|\mathcal{I}| = 84$ and $|\mathcal{J}| = 10$. We minimise F under the constraints $\eta > 0$ and $c_{D_H}(i) > 0$ using the Constrained Optimisation By Linear Approximation (COBYLA) algorithm [2, 3, 5]. The resulting parameter estimations are presented in Table 1.

Parameter	Value	Std.
$\gamma_H \mu_H^{-1}$	0.8852	0.0511
$\bar{x}_H$	1.458	0.4204
$\bar{x}_D$	-0.7041	0.2661

Table 1: Results of fitting the linear model defined by (16) to obtain the best fit values for $\eta = \gamma_H \mu_H^{-1}$, and thus means $\bar{x}_H$ and $\bar{x}_D$.

2.2 Inference for hospital model

In this section we present the inference equations and the observational model we derived in order to infer the parameters and initial conditions. The concept is as follows: we define variables that describe the data we have, in terms of the model compartment and parameters, and express the model to only be in terms of these new variables and the model parameters. This establishes what parameters we can identify and infer from the data.

2.2.1 Mathematical notation

The data we have to utilise is daily hospital admissions, daily hospital discharges, daily hospital bed occupancy and weekly non-hospital deaths. Note, we do not consider hospital deaths as we have already used this data to find the relationship on $\gamma_H \mu_H^{-1}$. In terms of the hospital data, considering (5), we know that $\dot{H} = H_{in} - H_{out} := admitted - (discharged + died)$ and thus

$$c_{Ad}(t) := \gamma_I \int_{t-1}^{t} I(s)\,\mathrm{d}s, \tag{17}$$

describes the daily hospital admissions, and

$$c_{Dis}(t) := \gamma_H \int_{t-1}^{t} H(s)\,\mathrm{d}s \tag{18}$$

describes the daily hospital discharges. Similarly, considering (6), we have that

$$c_{D_U}(t) := m_U \gamma_U \int_{t-1}^{t} U(s)\,\mathrm{d}s \tag{19}$$

describes the daily deaths outside of hospital. Note, for ease of notation we consider daily death data rather than weekly, and we have denoted (18) this way so as not to confuse with (10), since although it is the same data, the variables are being used in different settings. So far we have not introduced the hospital bed occupancy data, since this is described directly by $H(t)$ we will include this straight into the log-likelihood function to be fit directly to the data.

Before we derive the observational model we re-introduce the full model for ease of exposition, where we have omitted the deaths and recovered compartments (equations (6)-(9)), namely

$$\dot{S} = -\tilde{\beta}(U + I)S, \tag{20}$$
$$\dot{E} = \tilde{\beta}(U + I)S - \gamma_E E, \tag{21}$$
$$\dot{U} = p\gamma_E E - \gamma_U U, \tag{22}$$
$$\dot{I} = (1-p)\gamma_E E - \gamma_I I, \tag{23}$$
$$\dot{H} = \gamma_I I - \tilde{\mu}_H \gamma_H H. \tag{24}$$

Here we have also denoted $\tilde{\beta} = \beta N^{-1}$ and $\tilde{\mu}_H = 1 + \mu_H \gamma_H^{-1}$ for ease of notation.

2.2.2 Observational Model

We first look to manipulate equations (20)–(24) to only be in terms of the compartments $U(t)$, $I(t)$ and $H(t)$ and their derivatives so that an easy application of calculus gives us the reduced equations in terms of c_{Ad}, c_{Dis} and c_{D_U}. Since we have three variables, we look to derive three equations in terms of U, I and H.

One notices that (24) is already in terms of the required compartments. We can use (23) to gain E in terms of the required compartments, namely

$$E = \frac{\dot{I} + \gamma_I I}{(1-p)\gamma_E}, \tag{25}$$

which we can then use in (22) to gain our second necessary equation. To gain the third equation we utilise (25) to manipulate (20) and (21) to be in terms of the required compartments, which leads us to the following system of equations

$$\dot{H} = \gamma_I I - \tilde{\mu}_H \gamma_H H, \tag{26}$$

$$\dot{U} = \frac{p}{1-p}(\dot{I} + \gamma_I I) - \gamma_U U, \tag{27}$$

$$\frac{\dddot{I} + \gamma_I \ddot{I}}{(1-p)\gamma_E} + \frac{\ddot{I} + \gamma_I \dot{I}}{1-p} = \left(\frac{\ddot{I} + \gamma_I \dot{I}}{(1-p)\gamma_E} + \frac{\dot{I} + \gamma_I I}{1-p} \right) \frac{\dot{U} + \dot{I} - \tilde{\beta}(U+I)^2}{U+I}. \tag{28}$$

For ease of notation, let us denote by $x := \dot{c}_{Ad}$, $y := \dot{c}_{D_U}$ and $z := \dot{c}_{Dis}$. Thus, using (17)–(19) in (26)–(28), the observational model for (20)–(24) is

$$\begin{aligned}
\dddot{x} &= (\ddot{x} + (\gamma_I + \gamma_E)\dot{x} + \gamma_I\,\gamma_E\,x) \left(\frac{x}{\gamma_I} + \frac{y}{m_U \gamma_U} \right)^{-1} \\
&\quad \times \left(\frac{1}{1-p}\frac{\dot{x}}{\gamma_I} + \frac{p}{1-p}x - \frac{y}{m_U} - \tilde{\beta}\left(\frac{x}{\gamma_I} + \frac{y}{m_U \gamma_U} \right)^2 \right) - (\gamma_I + \gamma_E)\ddot{x} - \gamma_I\,\gamma_E\,\dot{x}, && (29) \\
\dot{y} &= \frac{m_U \gamma_U\, p}{1-p}\left(\frac{\dot{x}}{\gamma_I} + x \right) - \gamma_U y, && (30) \\
\dot{z} &= \gamma_H x - \gamma_H \tilde{\mu}_H z. && (31)
\end{aligned}$$

Note, the observational model involves all the parameters so given enough data points, and since the Jacobian is invertible, this implies structural identifiability.

2.2.3 Inference

Now that we have the observational model of (20)–(24) in terms of the data, we can proceed to fit the data and gain the inferred parameter values by minimising a log-likelihood function. In a similar manner to Section 2.1 we denote the set of daily hospital admissions data as C_{Ad}, with each data point denoted as $C_{Ad,i}$, for $i = 1, \ldots, 84$. Similarly, we denote the set of daily hospital discharge data as C_{Dis}, with each data point denoted as $C_{Dis,i}$, for $i = 1, \ldots, 84$. We denote the set of daily hospital bed occupancy data as C_{HO}, with each data point denoted as $C_{HO,i}$, for $i = 1, \ldots, 84$. Finally, we denote the set of weekly death data outside of hospital as $C^w_{D_U}$, with each data point denoted as $C^w_{D_U,j}$, for $j = 14, \ldots, 23$. Assuming that the data is collected subject to centered Gaussian errors, scaling the mortality data error to account for weekly data, we have the following negative log-likelihood function to minimise

$$\begin{aligned}
F(c_{Ad}, c_{Dis}, H, c_{D_U}; C_{Ad}, C_{Dis}, C_{HO}, C_{D_U}) &:= \frac{1}{|\mathcal{I}|}\sum_{i=1}^{84} (c_{Ad}(i) - C_{Ad,i})^2 + \frac{1}{|\mathcal{I}|}\sum_{i=1}^{84} (c_{Dis}(i) - C_{Dis,i})^2 \\
&+ \frac{w_1}{|\mathcal{I}|}\sum_{i=1}^{84} (H(i-1) - C_{HO,i})^2 + \frac{1}{7|\mathcal{J}|}\sum_{j=14}^{23} \left(\sum_{k=0}^{6} c_{D_U}(t_j - k) - C^w_{D_U,j} \right)^2, \qquad (32)
\end{aligned}$$

where $\mathcal{I}$ and $\mathcal{J}$ are defined as before. We have introduced the weight $w_1 = 10^{-1}$ so that the data is comparable, i.e. so that the magnitude of the daily hospital occupancy is of the same magnitude as the daily hospital admissions and discharge data.

Without any constraints, (32) has multiple local minimisers. We therefore impose two physically relevant constraints based on the following assumptions. First, we assume that the population within the model, excluding the recovered and death compartments, is between 90% and 100% of the total population N that we consider. This reflects the fact that an unknown fraction of the population is already immune or are not susceptible, due to the shielding programmes in place, for example. Second, we assume that the effective reproductive number, defined as

$$\mathcal{R}_t := \frac{S_0}{N}\mathcal{R}_0, \tag{33}$$

at the beginning on the simulation is less than 1 but greater than 0.6. This reflects the effect of the lockdown on the population dynamics and avoids non-feasible parameters that involve very high infection rates leading to close to 100% of infections in a short period of time, which is unrealistic in the time period of the data we are considering. Note, $\mathcal{R}_0$ is the basic reproductive number and is equivalent to $\mathcal{R}_t$ when $S_0 = N$, which is typically comparable to the very beginning of an epidemic, i.e. when there are very few individuals infected. This is not necessarily the case here as we don't know how many individuals have already been infected at the beginning of the lockdown period, when our first bit of data is, and thus the beginning of our simulation, hence why we explicitly differentiate between notation here.

Using the method of next-generation matrices described in [4], we obtain the following expression for the basic reproductive number

$$\mathcal{R}_0 := \beta \left(\frac{p}{\gamma_U} + \frac{1-p}{\gamma_I} \right). \tag{34}$$

Utilising a similar method as in Section 2.2.2, one can obtain the initial conditions S_0, E_0, U_0 and I_0 in terms of the data, whereby we already have H_0 since $H_0 = C_{HO,1}$ by definition. Including the constraints, the negative log-likelihood then reads

$$\begin{aligned} G(c_{Ad}, c_{Dis}, H, c_{D_U}, S_0, E_0, U_0, I_0; C_{Ad}, C_{Dis}, C_{HO}, C_{D_U}, N) \\ := \frac{1}{|\mathcal{I}|} \sum_{i=1}^{84} (c_{Ad}(i) - C_{Ad,i})^2 + \frac{1}{|\mathcal{I}|} \sum_{i=1}^{84} (c_{Dis}(i) - C_{Dis,i})^2 + \frac{w_1}{|\mathcal{I}|} \sum_{i=1}^{84} (H(i-1) - C_{HO,i})^2 \\ + \frac{1}{7|\mathcal{J}|} \sum_{j=14}^{23} \left(\sum_{k=0}^{6} c_{D_U}(t_j - k) - C_{D_U,j} \right)^2 \\ - w_2(1 - \mathcal{R}_t)(\mathcal{R}_t - 0.6) - w_3(N - N_0)(N_0 - 0.9N), \end{aligned} \tag{35}$$

where, for ease of notation, we have left the initial conditions and effective reproductive number in their original notation. To aid clarity in notation, we have introduced $N_0 = S_0 + E_0 + U_0 + I_0 + H_0$. For the simulation, we took the weights $w_2 = 10^2$ and $w_2 = 10^{-9}$ to make all the terms of the log-likelihood comparable. We minimise (35) using the limited memory Broyden–Fletcher–Goldfarb–Shanno algorithm with box constraints (L-BFGS-B) [1, 5, 6]. The box constraints were used to ensure positivity of the relevant parameters as well as positivity of the initial conditions.

2.3 Robustness analysis of parameters

Due to the size of the model along with the number of parameters, we don't consider a large-scale investigation into parameter sensitivity but conduct a rudimentary investigation. In order to measure the sensitivity of each parameter we calculate the change in the log-likelihood (in %) for a change in each of the parameters independently, i.e. we only change one parameter at a time. The optimal parameters (and thus chosen parameters in **Table 1** in the manuscript) make the smallest mismatch, i.e. the smallest output of the log-likelihood, of all the parameter values considered in the minimisation algorithm, and so we calculate the relative percentage change using the perturbed parameter from the optimal parameters. We note here that the observed value from the log-likelihood will never be zero due to the error in the data. We increase and decrease each of the fitted parameters by up to 1% from its optimal value and demonstrate the change in the log-likelihood in Table 2. As one can see, γ_U and m_U are not sensitive to changes at all but seemingly β, γ_E, p, γ_I and γ_H are. We postulate that this is in fact a structural issue of the model and the data rather than a parameter issue. If all the other parameters are fixed, both p and γ_E control the number of individuals we see in each of the branches describing U or I. We have data describing the number of individuals in the I branch, using hospital capacity data, and so both these parameters are actually very well characterised by the data, and thus any change in their values, with all the other parameters fixed, produces a large mismatch. Similarly, with p and γ_E fixed, γ_I controls the number of individuals moving from I to H and γ_H controls the number of individuals leaving H, and again, since we have the hospital admissions, occupancy and discharge data available, this value is also well characterised by the data. We also postulate that γ_U

and m_U are not as sensitive as the other parameters due to the lack of death data. It is expected that β is sensitive given that it is the main driving force behind generating infections in the model. These assertions are backed up by the results in Table 2 and Figure 2. For these results we took 30-50 data points and fitted the parameters to the data. One can see that γ_E, p and γ_I have a very small range within the different means, and they have a small range of standard deviations. Concentrating on the standard deviations, this means that the parameters would mostly not even be changing by 0.01% demonstrating that the actual fitting of the data is not sensitive at all. Qualitatively, changing each of the parameters by 1% results in an almost identical forecasting picture for the majority of the parameters, as can be seen in Figure 3. The reason that p looks largely different is due to the fact that p is much larger in value than the other parameters and so a 1% shift results in a larger range of values. For ease of demonstration we only demonstrate the hospital bed occupancy.

Parameter	0.01%	0.1%	1%
β	0.0019%	0.0186%	0.1876%
γ_E	0.0057%	0.0566%	0.5711%
p	0.0101%	0.1029%	1.244%
γ_U	0.0001%	0.0008%	0.0076%
γ_I	0.0045%	0.0447%	0.4521%
γ_H	0.0110%	0.1101%	1.113%
m_U	0.0007%	0.0072%	0.0724%

(a) Log-likelihood test.

Mean Range	Std. Range
[0.1457, 0.1540]	[0.0043, 0.0066]
[0.2141, 0.2142]	[6.25×10^{-6}, 1.26×10^{-5}]
[0.9271, 0.9275]	[2.34×10^{-6}, 4.39×10^{-6}]
[0.1980, 0.2074]	[0.0151, 0.0188]
[0.1587, 0.1588]	[4.74×10^{-6}, 9.40×10^{-6}]
[0.0533, 0.0594]	[0.0017, 0.0069]
[0.0249, 0.0262]	[4.70×10^{-5}, 8.25×10^{-5}]

(b) Parameter fits from using 30-50 data points.

Table 2: Results of the parameter robustness test.

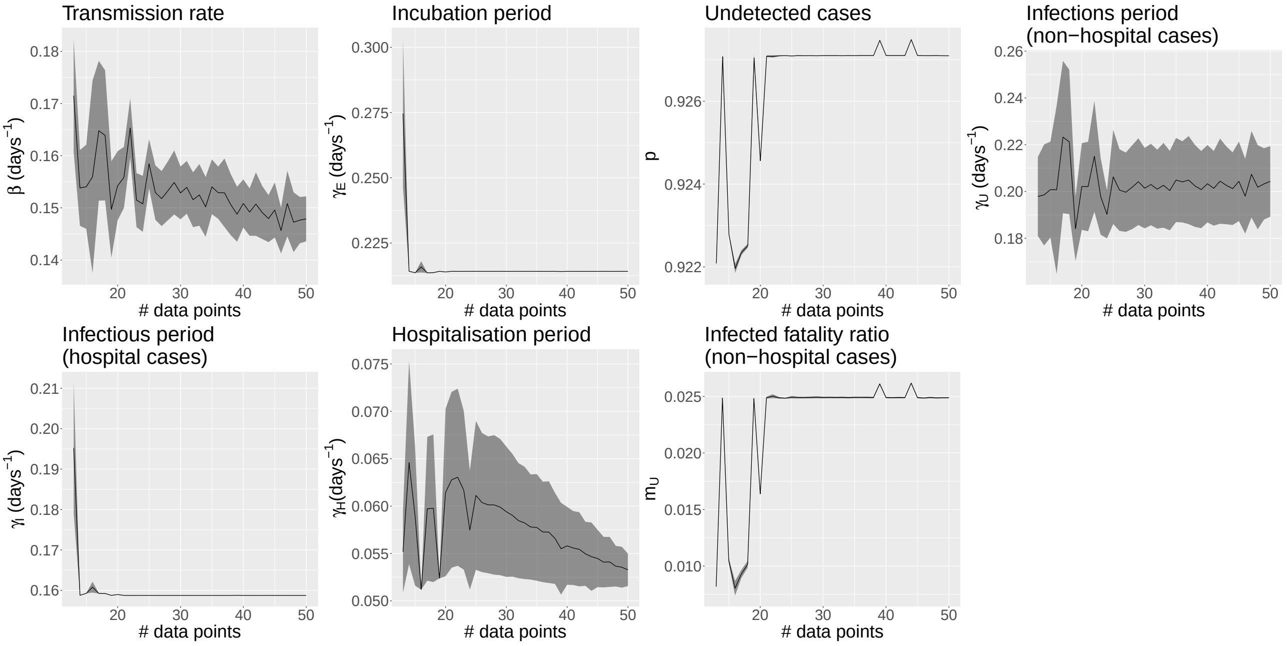

Figure 2: Parameter fits from using 30-80 data points.

References

[1] SciPy Community. fmin_l_bfgs_b. `https://docs.scipy.org/doc/scipy/reference/generated/scipy.optimize.fmin_l_bfgs_b.html`.

[2] SciPy Community. minimize(method='cobyla'). `https://docs.scipy.org/doc/scipy/reference/optimize.minimize-cobyla.html`.

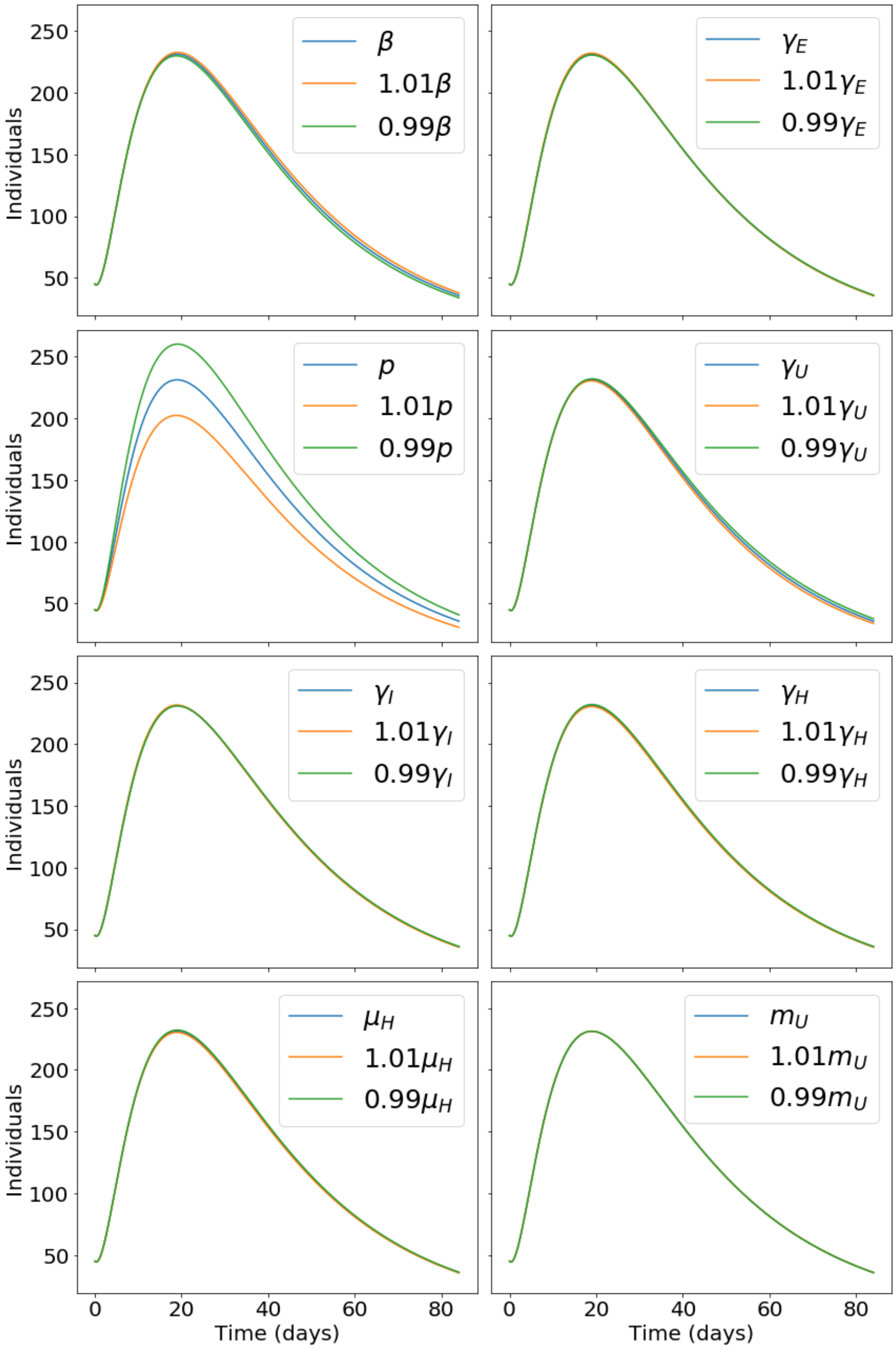

Figure 3: Parameter robustness test: beds occupied in hospitals.

[3] Andrew R Conn, Katya Scheinberg, and Luis N Vicente. *Introduction to derivative-free optimization*. SIAM, 2009.

[4] Odo Diekmann, JAP Heesterbeek, and Michael G Roberts. The construction of next-generation matrices for compartmental epidemic models. *Journal of the Royal Society Interface*, 7(47):873–885, 2010.

[5] Roger Fletcher. *Practical methods of optimization*. John Wiley & Sons, 2013.

[6] Robert Malouf. A comparison of algorithms for maximum entropy parameter estimation. In *COLING-02: The 6th Conference on Natural Language Learning 2002 (CoNLL-2002)*, 2002.
